# Beyond the crisis: Tracking chronic neuropathic pain in sickle cell disease using *Douleur Neuropathique* 4 and PainDETECT questionnaires

**DOI:** 10.1016/j.htct.2025.106231

**Published:** 2025-12-12

**Authors:** Camila Freitas de Andrade Rodrigues, Thiago Alves Rodrigues, Pedro Igor de Sousa Rios, Isabelle Nunes Costa, Bruno Feres de Souza, João Batista Santos Garcia

**Affiliations:** Universidade Federal do Maranhão (UFMA), São Luís, MA, Brazil

**Keywords:** Sickle cell disease, Neuropathic pain, Chronic pain, PainDETECT, *Douleur Neuropathique* 4

## Abstract

**Background:**

Neuropathic pain represents a complex and often underdetected component of the pain spectrum in Sickle Cell Disease, particularly among individuals with chronic or treatment-resistant symptoms. Despite its clinical relevance, neuropathic pain is not routinely screened for in hematology practice, where pain is frequently attributed solely to vaso-occlusive mechanisms.

**Method:**

A cross-sectional study was conducted with 214 individuals diagnosed with Sickle Cell Disease at a hematology referral center in northeastern Brazil. Two validated instruments, *Douleur Neuropathique* 4 and PainDETECT were utilized to screen for neuropathic pain. Clinical and demographic data were collected, and the correlation between the instruments was assessed using Pearson’s coefficient.

**Results:**

The *Douleur Neuropathique* 4 tool identified neuropathic pain in 29 % of participants. PainDETECT indicated 8.4 %, which increased to 22 % when including uncertain-range scores. The correlation between the two tools was strong (*r* = 0.87). Neuropathic pain was more prevalent among older individuals, those who reported recurrent painful episodes in the past year (p-value <0.001), and those with recent opioid use (p-value = 0.042). Sensory descriptors such as tingling, numbness, and electric shock sensations were commonly reported.

**Conclusion:**

The combined use of *Douleur Neuropathique* 4 and PainDETECT, both of which are quick and simple to administer, proved to be a complementary strategy for identifying neuropathic pain, with each instrument capturing distinct features. Incorporating this approach into hematology care may facilitate the detection of pain profiles beyond vaso-occlusion and support more individualized treatment decisions.

## Introduction

Pain is one of the most common manifestations of Sickle Cell Disease (SCD) and often serves as the primary reason for hospitalizations within this population. The traditional explanation links these painful crises to vaso-occlusive episodes, primarily involving inflammatory and nociceptive mechanisms. However, recent studies underscore the complexity of this phenomenon, suggesting that some patients may experience pain with neuropathic characteristics [1,2].

Simply put, nociceptive pain is understood to arise from the activation of peripheral receptors by damaging stimuli, as seen in cases of tissue hypoxia. In contrast, neuropathic pain (NP) is associated with lesions or dysfunctions of the somatosensory nervous system, exhibiting symptoms such as paresthesia, burning pain, or the sensation of electric shock. These represent two distinct entities, both in terms of pathophysiology and therapeutic approach, although this distinction is still inadequately incorporated into the clinical practice of many professionals [3].

In many patients with SCD, pain is not limited to acute vaso-occlusive episodes. It may also include symptoms such as allodynia, persistent limb discomfort, tingling, burning, or pain triggered by light touch, complaints that are typically associated with a neuropathic component. These cases challenge the traditional perspective on sickle cell pain and suggest that neuroimmune mechanisms may also play a role, rather than pain being solely attributed to vascular occlusion. Evidence has demonstrated that the activation of glial cells, especially microglia and astrocytes, can lead to the release of pro-inflammatory cytokines such as Tumor necrosis factor-alpha, interleukin (IL)-1β, and IL-6. These mediators enhance neuronal excitability and promote abnormal pain signaling, contributing to central sensitization and the persistence of pain even in the absence of acute tissue injury [3,4].

Although neuropathic mechanisms have been described in SCD, this type of pain often remains unrecognized in clinical practice. It is estimated that between 25 % and 40 % of individuals with SCD suffer from NP, many of them without proper identification or treatment [4].

Tools like the *Douleur Neuropathique* 4 (DN4) and PainDETECT questionnaires are commonly used to assess NP across various populations. Both are simple and quick to administer, but they differ in structure and focus. The DN4 combines symptom descriptors with physical examination findings, while the PainDETECT is entirely self-administered and emphasizes sensory descriptors and pain patterns [5,6]. Few studies have compared these instruments in patients with SCD, making it challenging to standardize diagnoses and identify more effective therapeutic approaches.

Although pain is a primary concern for patients with SCD in clinical contexts, the potential for neuropathic mechanisms is often overlooked in many settings, particularly outside pain specialties. This oversight is not necessarily due to a lack of knowledge but may reflect the prioritization of other clinical manifestations that are also prevalent in the hematology practice. Increased awareness of the different pain mechanisms in this patient group could directly impact therapeutic management and improve quality of life. In light of this, this study aimed to evaluate the prevalence of NP in SCD patients by utilizing the DN4 and PainDETECT questionnaires. Additionally, the study sought to identify clinical characteristics related to the presence of NP and to compare the diagnostic findings provided by each instrument.

## Material and methods

### Study design and setting

This is a cross-sectional study conducted with patients diagnosed with SCD who are regularly followed at the Hematology and Hemotherapy Center of Maranhão (HEMOMAR), a state reference institution for hemoglobinopathies located in São Luís, Maranhão, Brazil. Data were collected between December 2022 and December 2024.

### Sample and inclusion and exclusion criteria

The sample was composed for convenience of patients who attended routine appointments during the study period. Individuals aged 14 years and older, of both genders, with a confirmed diagnosis of SCD by hemoglobin electrophoresis (via high-performance liquid chromatography) were included. Participants needed to be in adequate clinical, cognitive, and emotional condition to answer the interview and must have formally consented to participate. For participants under 18, an assent form was obtained in addition to informed consent from their legal guardians. Individuals experiencing a pain crisis at the time of the interview, pregnant women, patients with severe psychiatric disorders, or those with hearing or speech deficits were excluded.

### Data collection procedures

After the signing of the consent and assent forms, participants completed a structured questionnaire that collected sociodemographic data, including age, gender, skin color, education, city of origin, occupation, and family income, as well as clinical data such as age at diagnosis, hemoglobin genotype, number of hospitalizations, transfusions in the past 12 months, occurrence of pain crises in the past 12 months, and use of opioids. Routine laboratory data were obtained from medical records, which included hemoglobin levels, leukocyte count, platelet count, and hemoglobin electrophoresis.

### Instruments used

In this study, two validated instruments were used to screen for NP: the DN4 and the PainDETECT Questionnaires. The DN4 consists of ten items: seven related to self-reported sensory symptoms and three derived from physical examination. These items assess sensations such as burning, painful cold, electric shocks, tingling, pins-and-needles, numbness, and itching, as well as the presence of mild touch hypoesthesia, pinprick hypoesthesia, and allodynia. Each item is scored using a binary system (0 or 1), resulting in a total score that ranges from 0 to 10. A score of 4 or higher is considered indicative of probable NP. The Portuguese version of the DN4 has been validated, and its brevity and diagnostic accuracy make it suitable for use in outpatient hematology settings [5,7,8].

The PainDETECT Questionnaire is a self-administered tool originally developed for patients with chronic low back pain but has also been validated for other NP conditions. It consists of four main sections. The first section contains three items that assess current, average, and maximum pain intensity over the past four weeks, using a numerical scale from 0 to 10. The second section presents four temporal patterns of pain, while the third examines the presence and direction of pain irradiation illustrated on a body diagram. The fourth section includes seven sensory descriptors (burning, tingling, allodynia, pain attacks, temperature-evoked pain, numbness, and pressure pain), evaluated on a Likert scale from 0 (none) to 5 (very strong). The final score ranges from 0 to 38: scores of 19 or higher indicate probable NP; scores of 12 or lower suggest that this mechanism is unlikely; and scores between 13 and 18 represent a zone of diagnostic uncertainty. The questionnaire also includes a body diagram, where patients indicate the location and radiation of pain, and a panel of line graphs representing different temporal pain patterns (e.g., persistent, fluctuating, intermittent) to help the patient select the pattern that best characterizes their experience. Although these visual components are not included in the scoring algorithm, they provide useful clinical information. The Portuguese version of the PainDETECT Questionnaire was adapted and validated, demonstrating good diagnostic accuracy. Similar to the DN4, its ease of use and applicability in outpatient non-acute contexts make it a valuable instrument for routine pain screening in patients with SCD [6,9].

All assessments were conducted in an outpatient setting, and only patients in stable clinical conditions were included. Individuals experiencing vaso-occlusive crises at the time of evaluation were excluded, as acute pain episodes can distort symptom reporting and introduce bias in responses to both the DN4 and PainDETECT questionnaires. This approach ensured that participants' answers reflected their typical pain experience rather than transient exacerbations, and minimized the risk of cognitive or emotional interference associated with acute stress.

## Statistical analysis

Categorical variables were analyzed using Fisher's exact test based on their suitability. For continuous variables, the Mann-Whitney test was applied. Binary logistic regression was performed to estimate adjusted odds ratios (OR) with 95 % confidence intervals (95 % CI). The significance level was set at 5 % (p-value <0.05). The analyses were carried out using appropriate statistical software.

## Ethical aspects

The Research Ethics Committee at the University Hospital of the Federal University of Maranhão (HUUFMA) approved the study under opinion CAAE 51,633,821.1.0000.5086, in accordance with the ethical guidelines outlined in Resolution 466/2012 by the National Health Council.

## Results

This study evaluated 214 patients with SCD who were regularly monitored at the Hematology and Hemotherapy Center of Maranhão and completed questionnaires to identify NP.

Among the participants, there was a predominance of females: 125 (58 %) compared to 89 males (42 %). The average age was 26.2 years, with 28 % aged between 14 and 18 years old. Regarding self-identified race, 24 % identified themselves as Black, while 76 % identified themselves as non-Black. Approximately 14 % had an income of less than one minimum wage; 78 % earned between one and two salaries; and 8 % earned between three and five. About 49 % reported receiving disability benefits. In terms of education, 24 % had low education levels (illiterate to incomplete primary education), 61 % had intermediate education (completed primary to secondary education), and 15 % had completed or incomplete higher education. Although the study took place in the capital, approximately 69 % of the participants lived in the interior of the state of Maranhão.

The NP questionnaires revealed the following findings: DN4 was positive for 62 patients (29 %), with an average score of 5.64. PainDETECT was positive for 18 patients (8.4 %), with an average score of 21.8 points. When considering individuals with intermediate PainDETECT scores (between 13 and 18 points, the diagnostic uncertainty zone), a total of 48 patients (22 %) exhibited possible NP, with an average score of 18 points.

For analytical purposes, participants were classified according to the DN4 questionnaire (score ≥4), which was used as the primary reference instrument for the group comparisons presented in Table 1, Table 2. The PainDETECT questionnaire was applied as a complementary and comparative tool to analyze its correlation and agreement with the primary reference instrument.

**Table 1:** Demographic data of the patients included in the study.

Variable	n	% of total ( %)	No neuropathic pain* n = 152 ( %)	With neuropathic pain* n = 62 ( %)	OR	95 % CI	p-value	
Age group								
Between 14 and 18 years old	60	28	50 (33)	10 (16)	—	—	—	
Between 19 and 34 years old	99	46	69 (45)	30 (48)	1.61	0.66–4.11	0.3	
Over 34 years old	55	26	33 (22)	22 (35)	2.91	1.15–7.74	0.027	
Gender								
Female	125	58	87 (57)	38 (61)	—	—	—	
Male	89	42	65 (43)	24 (39)	1.12	0.57–2.19	0.7	
Race/skin color								
Non-black	162	76	121 (80)	41 (66)	—	—		
Black	52	24	31 (20)	21 (34)	1.85	0.92–3.71	0.083	
Education								
Low	52	24	38 (25)	14 (23)	—	—	—	
Average	131	61	93 (61)	38 (61)	1.36	0.61–3.18	0.5	
High	31	15	21 (14)	10 (16)	2.27	0.74–7.08	0.2	
Residence								
São Luís (Capital)	67	31	42 (28)	25 (40)	—	—	—	
Other	147	69	110 (72)	37 (60)	0.63	0.32–1.23	0.2	
Occupation								
Household/unemployed	62	29	44 (29)	18 (30)	—	—	—	
Paid work	38	18	24 (16)	14 (22)	1.06	0.39–2.86	>0.9	
Disability benefit	105	49	76 (50)	29 (47)	0.68	0.31–1.48	0.3	
Retired	9	4	8 (5)	1 (1)	0.15	0.01–1.07	0.10	
Family income								
<1 minimum wage	29	14	17 (11)	12 (20)	-	-		
1–2 minimum wages	166	78	120 (79)	46 (74)	0.56	0.23–1.39	0.2	
3–5 minimum wages	19	8	15 (9.9)	4 (6)	0.23	0.05–0.99	0.058	

⁎ according to DN4; OR: odds ratio adjusted by binomial logistic regression; 95 % CI: 95 % confidence interval; p-value <0.05 = statistically significant.

**Table 2:** Clinical data on sickle cell disease of the patients included in the study.

Variable	n	% of total	No neuropathic pain* n = 152 ( %)	With neuropathic pain* n = 62 ( %)	OR	95 % CI	p-value
Age at diagnosis							
<1 year old	85	40	65 (43)	20 (32)	—	—	—
1 - 5 years old	33	15	26 (17)	7 (11)	0.72	0.24–1.96	0.5
6 - 15 years old	49	23	35 (23)	14 (23)	0.98	0.40–2.35	>0.9
>15 years old	47	22	26 (17)	21 (34)	1.79	0.70–4.58	0.2
Subtype							
Hb SC	30	14	17 (11)	13 (21)	1.00	1.00–1.00	>0.9
Hb Sβ^0^	22	10	15 (10)	7 (11)	0.55	0.15–1.89	0.3
Hb SS	162	76	120 (79)	42 (68)	0.51	0.22–1.19	0.11
Pain crises in 12 months							
None	50	23	45 (30)	5 (8)	—	—	—
<3 episodes	76	35	61 (40)	15 (24)	2.27	0.79–7.61	0.15
≥3 episodes	88	42	46 (30)	42 (68)	8.12	3.07–25.9	<0.001
Opioid use in 12 months							
Yes	99	46	65 (43)	34 (55)	—	—	—
No	115	54	85 (56)	30 (48)	0.51	0.27–0.97	0.042
Hospitalizations in 12 months							
<3	189	88	137 (90)	52 (84)	—	—	—
≥3	25	12	15 (10)	10 (16)	1.65	0.66–4.03	0.3
Transfusions in 12 months							
<10 units	56	26	36 (24)	20 (32)	—	—	—
≥10 units	13	6	6 (4)	7 (11)	2.47	0.67–9.49	0.2
None	145	68	110 (72)	35 (56)	0.55	0.27–1.14	0.10

⁎ according to DN4; OR: odds ratio adjusted by binomial logistic regression; 95 % CI: 95 % confidence interval; p-value <0.05 = statistically significant.

Regarding genotype, 76 % were hemoglobin (Hb) SS, 14 % were Hb SC, and 10 % were Hb Sβ⁰. Neonatal diagnosis through the heel prick test had identified 40 % of cases; 15 % were diagnosed by age five, 23 % between the ages of 6 and 15, and 22 % only after age 15.

Approximately 77 % of patients reported experiencing two or more episodes of pain in the past 12 months. This proportion was higher among those with NP (68 % compared to 30 %), indicating statistical significance (p-value <0.001). Opioid use was more common among individuals with NP (55 %; p-value = 0.042). The frequency of hospitalizations and transfusions in the last year was also elevated in this group, although it did not reach statistical significance.

Laboratory data, including hemoglobin concentration, total leukocyte and platelet counts, and percentage of Hb S, showed no differences between the groups, as shown in Table 3.

**Table 3:** Laboratory data on the patients included in the study.

Variable	Value	No neuropathic pain* n = 152	With neuropathic pain* n = 62	p-value
Hemoglobin (g/dL)	9.1	9.00 (7.80–10.25)	9.20 (8.00–10.20)	0.5
Leukocytes (/mm^3^)	8072.50	7980 (5780–10,980)	8165 (5800–10,750)	0.2
Platelets (x 10^3^/mm^3^)	327	344 (257–462)	310 (212–419)	0.3
Hemoglobin S ( %)	78	79 (70–86)	77 (53–86)	0.2

⁎ Values are presented as Median (Q1; Q3).

A total of 128 patients (60 %) reported using hydroxyurea. This variable showed no statistically significant difference when comparing the groups with and without NP (p-value = 0.4).

**Fig. 1:**
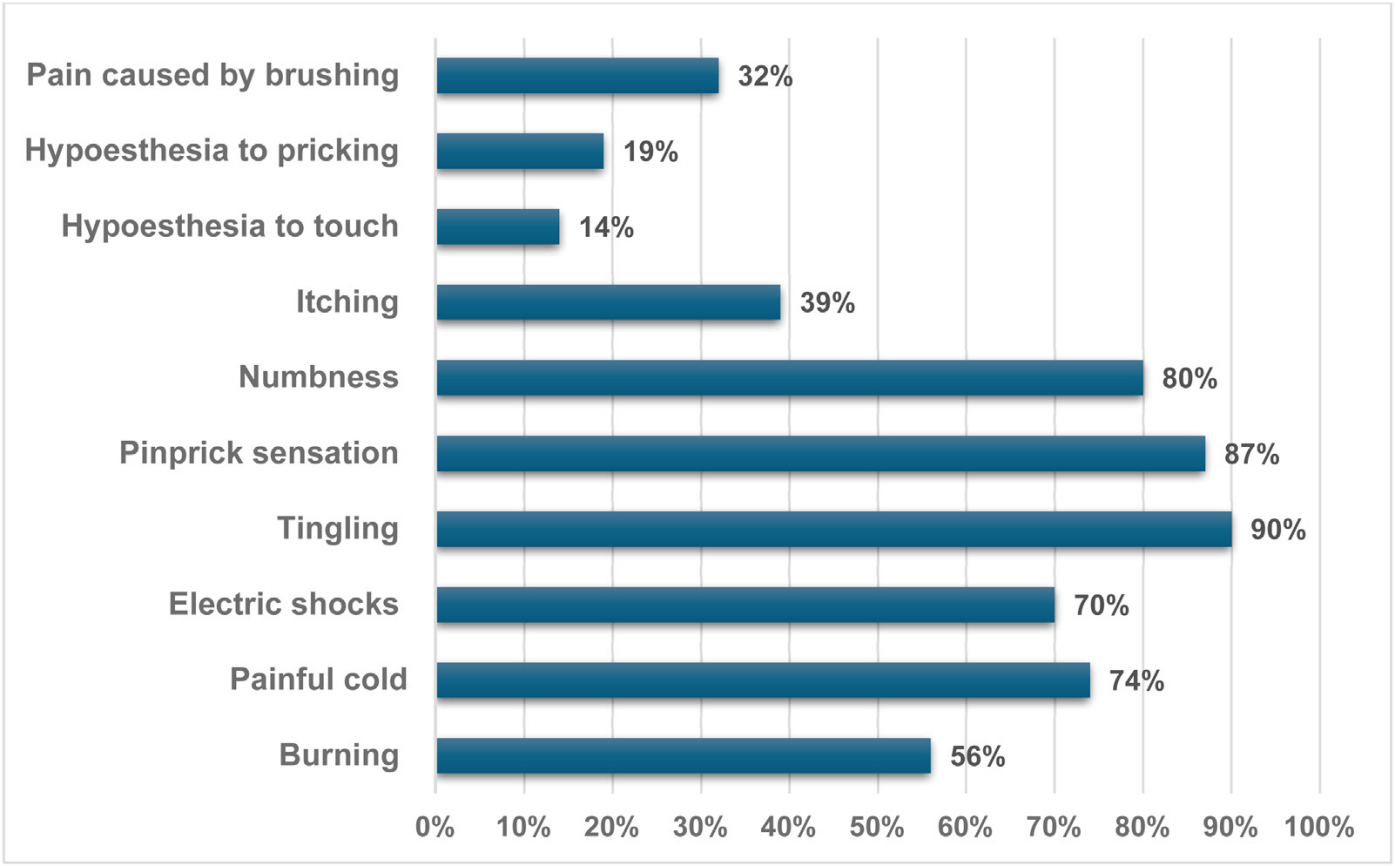
The most common complaints identified using the DN4 questionnaire in patients with neuropathic pain related to sickle cell disease (n = 62).

Among patients with NP indicated by DN4, the most common symptoms were tingling (90 %), a pinprick sensation (87 %), numbness (80 %), painful cold (74 %), electric shocks (70 %), and burning (56 %). Other symptoms reported included itching (39 %), pain caused by brushing (32 %), hypoesthesia to pricking (19 %), and hypoesthesia to touch (14 %) (Figure 1).

The PainDETECT questionnaire also enabled the capture of various aspects of the pain reported by patients. Among those diagnosed with DN, this instrument indicated that the average pain at the time of the interview was 3.2 (on a scale of 0 to 10). When asked about the pain experienced over the last four weeks, the average score was 6.4, with the most intense peak reported being on average 8.9.

**Fig. 2:**
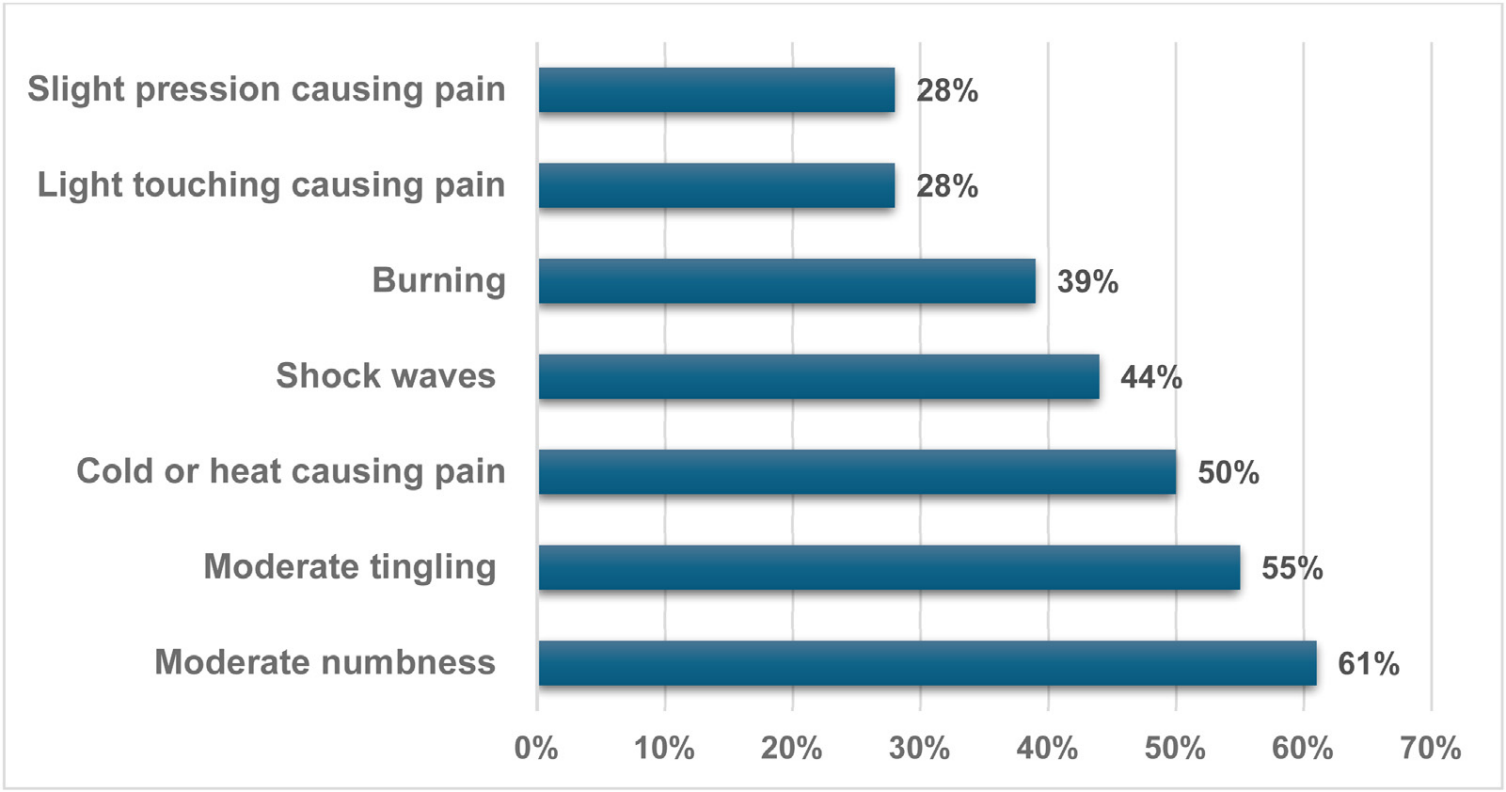
The most common complaints identified using the PainDETECT questionnaire in patients with neuropathic pain related to sickle cell disease (n = 18).

Another aspect of PainDETECT involved depicting the pain pattern using a graphic image that best represents it. It was observed that 40 % of those with ne NP described frequent pain crises occurring at intervals, while 33 % reported pain crises without complaints during those times. Additionally, 27 % noted constant pain accompanied by crises, while none described constant pain with slight variations. Furthermore, a significant 94 % of patients with NP characterized their pain as radiating. Among the most notable sensations were moderate numbness in 61 %, moderate tingling in 55 %, and temperature (cold or heat) causing occasional pain in 50 %. Shock waves were also reported by 44 % of patients, adding to the variety of pain experiences (Figure 2).

**Fig. 3:**
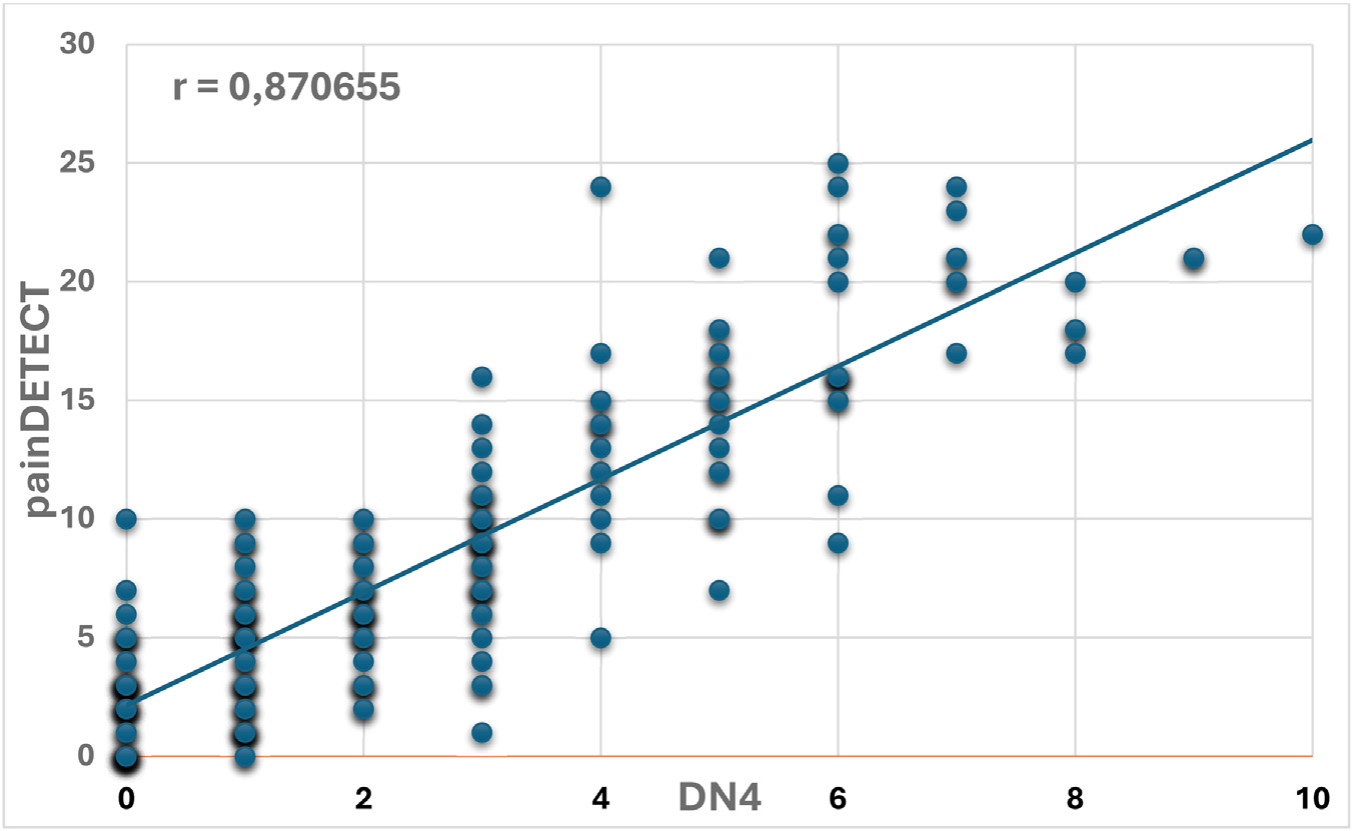
Correlation between DN4 and PainDETECT questionnaires for detecting neuropathic pain in sickle cell disease.

The scores obtained from the DN4 and PainDETECT instruments were compared using Pearson’s coefficient (*r* = 0.870655). This indicates a strong positive correlation, confirming the high agreement in identifying and quantifying NP in this population. This finding provides reassurance regarding the reliability of these instruments, despite the differences in prevalence observed when each tool is applied independently (Figure 3).

## Discussion

NP remains underrecognized in SCD, either due to limited clinician awareness or the considerable variability in symptom presentation. This lack of recognition often creates the perception that these patients are particularly challenging to manage [1,10]. This investigation aimed to explore this dimension by applying two validated screening tools, DN4 and PainDETECT, to assess how each performs in identifying neuropathic features and where their findings converge or diverge.

Interest in diagnosing NP in individuals with SCD has increased in recent years, with studies indicating its presence in up to 40 % of patients, a rate that exceeds that observed in other neuropathic conditions, such as painful diabetic neuropathy [11,12]. In the current study, the prevalence of NP was 29 % according to the DN4 assessment and 8.4 % with PainDETECT; including patients with scores in the uncertain range raised this figure to 22 %. These differences reflect findings from previous studies and illustrate how diagnostic criteria and instrument characteristics can influence detection rates [1]. Notably, the prevalence of NP observed in the present sample regardless of the instrument used was comparable to or higher than general population estimates that typically range from 6–10 % [13]. This finding reinforces the need to improve both recognition and management of NP in individuals with SCD.

A study conducted in Bahia, Brazil, using the DN4 questionnaire, found that NP was present in 69 of 100 individuals with SCD. However, many of those patients also reported experiencing depression and anxiety, which may have influenced their reporting of symptoms [14].

In another study, patients with SCD from Brazil and France were assessed using PainDETECT via telephone. In Brazil, 55 % showed a likely neuropathic component, 23 % were negative, and 22 % were uncertain. Similarly, in France, results indicated 51 % positive, 29 % negative, and 20 % unclear, with all participants reporting constant pain. Opioid use was reported by 62 % of Brazilian patients and 32 % of French patients. These findings highlight the challenge of identifying pain subtypes and the implications of inadequate treatment [10].

Other instruments for identifying NP can also be used in this context. For example, the Leeds Assessment of Neuropathic Symptoms and Signs Pain Scale (LANSS tool) identified NP in 25 % of 56 SCD patients [15], emphasizing the diversity of pain mechanisms in SCD and the need for more precise diagnostic approaches [16].

The present study identified a strong correlation between DN4 and PainDETECT (*r* = 0.87), suggesting that both instruments capture overlapping features of NP despite differing prevalence estimates. The DN4 emphasizes classical neuropathic descriptors, whereas PainDETECT also incorporates pain intensity and temporal patterns, potentially reflecting central sensitization. Although previous studies have utilized different instruments in individuals with SCD, few have examined their concurrent use through direct statistical comparison [17,18]. These findings contribute novel evidence by highlighting the complementary strengths of DN4 and PainDETECT in capturing NP characteristics in a stable outpatient population with SCD.

The DN4 assessment also includes a brief physical examination, which may enhance its diagnostic utility in clinical settings. PainDETECT features a gray area (13–18 points) that does not confirm a diagnosis but can still raise clinical suspicion. The absence of a disease-specific questionnaire for NP in SCD present an opportunity to utilize general screening tools. However, instruments like DN4 and PainDETECT serve as more than just general screening tools; they are easy to administer, require no more than five minutes, and can provide valuable insights to guide clinical management, including in hematology outpatient settings. Their integration by hematologists into chronic pain evaluations may significantly enhance the care of patients with complex or refractory pain presentations. Their potential as practical diagnostic tools offers hope for the future management of NP in SCD.

In this study, older patients were more likely to experience NP. This aligns with the broader literature on pain, which indicates that aging is associated with an increased risk of NP. Neuroplasticity may alter pain perception over time, and repeated vaso-occlusive episodes can lead to cumulative nerve damage, chronic nociceptor activation, and increased sensitivity [2,18].

While some previous studies suggest a higher prevalence of NP in women, the findings of this study did not indicate a sex difference, which aligns with other reports [15,18]. This highlights the need for ongoing research to identify clinical predictors of NP in SCD.

Among patients identified with NP in this sample, 94 % reported radiating pain. Symptoms such as tingling (90 %), pins-and-needles (87 %), and numbness (80 %) were common, as revealed by the DN4 tool. These findings support the hypothesis that NP in SCD may result from chronic ischemia or nerve injury, as previously reported in the literature [19]. Similar symptoms were also the most frequently observed in a study of 54 younger patients [20]. These data may enhance clinical interviews when specific tools are unavailable.

Another significant finding was the strong association between NP and frequent pain crises (p-value <0.001). Patients with NP experienced more episodes in the past year, suggesting a cycle in which repeated pain could lead to central sensitization, chronicity, and poorer function [21].

This study also found that 55 % of patients with NP had used opioids in the previous year, a significantly higher rate compared to those without NP (p-value = 0.0042). While opioids are often used to manage acute vaso-occlusive pain, they are less effective for NP and may even contribute to hyperalgesia. In these cases, alternative medications such as gabapentin (Neurontin)oids, tricyclic antidepressants, and serotonin-norepinephrine reuptake inhibitors might be more appropriate [22,23]. However, none of the patients identified with NP in this study were receiving any specific adjuvant treatment, including gabapentin (Neurontin)oids, at the time of data collection. This indicates that NP had not been previously diagnosed in these individuals and was only identified through the application of DN4 and PainDETECT during the study. This finding underscores the gap in clinical recognition and the potential of systematic screening tools to enhance more appropriate and individualized pain management strategies in patients with SCD [22,23].

This study has limitations. As a cross-sectional analysis, it cannot establish causality between NP and other variables. Additionally, because the tools used vary in sensitivity, some variation in diagnostic rates is expected. Nonetheless, the strong correlation between DN4 and PainDETECT supports the reliability of our findings. Further multicenter studies and the development of specific screening tools for SCD may enhance the detection and management of NP across diverse care settings.

## Conclusion

This study shows that NP is an important yet often overlooked aspect of the pain experience in individuals with SCD. Using NP and PainDETECT allowed us to identify various pain patterns and demonstrated agreement between the instruments. These simple and efficient tools could be valuable additions to the routine evaluation of patients, particularly those whose pain does not respond as expected to standard treatments. Incorporating this type of screening into hematology practice may help refine therapeutic choices and improve clinical outcomes. This highlights the need to adopt diagnostic approaches that better reflect the complexity of pain in SCD.

## Declaration of generative AI and AI-assisted technologies in the writing process

During the preparation of this work, the author(s) used ChatGPT 4.0 and Grammarly to enhance language and readability. After utilizing these tools, the author(s) reviewed and edited the content as needed and take full responsibility for the publication's content.

## Data availability statement

The data that support the findings of this study are available from the corresponding author upon reasonable request.

## Conflicts of interest

None.
